# Systematic assessment of benefits and risks: study protocol for a multi-criteria decision analysis using the Analytic Hierarchy Process for comparative effectiveness research

**DOI:** 10.12688/f1000research.2-160.v1

**Published:** 2013-07-24

**Authors:** Nisa M Maruthur, Susan Joy, James Dolan, Jodi B Segal, Hasan M Shihab, Sonal Singh

**Affiliations:** 1 Department of Medicine, The Johns Hopkins University School of Medicine, Baltimore MD, 21205, USA; 2 Department of Epidemiology, The Johns Hopkins University Bloomberg School of Public Health, Baltimore MD, 21205, USA; 3 The Welch Center for Prevention, Epidemiology, and Clinical Research, Baltimore MD, 21205, USA; 4 Department of Health Policy and Management, The Johns Hopkins University Bloomberg School of Public Health, Baltimore MD, 21205, USA; 5 Department of Public Health Sciences, University of Rochester School of Medicine and Dentistry, Rochester NY, 14642, USA

## Abstract

**Background**: Regulatory decision-making involves assessment of risks and benefits of medications at the time of approval or when relevant safety concerns arise with a medication. The Analytic Hierarchy Process (AHP) facilitates decision-making in complex situations involving tradeoffs by considering risks and benefits of alternatives. The AHP allows a more structured method of synthesizing and understanding evidence in the context of importance assigned to outcomes. Our objective is to evaluate the use of an AHP in a simulated committee setting selecting oral medications for type 2 diabetes.

**Methods: **This study protocol describes the AHP in five sequential steps using a small group of diabetes experts representing various clinical disciplines. The first step will involve defining the goal of the decision and developing the AHP model. In the next step, we will collect information about how well alternatives are expected to fulfill the decision criteria. In the third step, we will compare the ability of the alternatives to fulfill the criteria and judge the importance of eight criteria relative to the decision goal of the optimal medication choice for type 2 diabetes. We will use pairwise comparisons to sequentially compare the pairs of alternative options regarding their ability to fulfill the criteria. In the fourth step, the scales created in the third step will be combined to create a summary score indicating how well the alternatives met the decision goal. The resulting scores will be expressed as percentages and will indicate the alternative medications' relative abilities to fulfill the decision goal. The fifth step will consist of sensitivity analyses to explore the effects of changing the estimates. We will also conduct a cognitive interview and process evaluation.

**Discussion**: Multi-criteria decision analysis using the AHP will aid, support and enhance the ability of decision makers to make evidence-based informed decisions consistent with their values and preferences.

## Background

Regulatory decision-making involves assessment of the risks and benefits of medications at the time of approval or when relevant safety concerns arise with a medication. The objectives of regulatory decisions are to determine whether a particular drug is safe and effective for use in the population at a specific dose for a particular indication. With increasing pressure on government agencies to improve transparency, the Food and Drug Administration (FDA) is making significant changes to make its processes and decisions more transparent to industry stakeholders and consumers ( http://www.fda.gov/AboutFDA/Transparency/TransparencyInitiative/default.htm). This has included initiatives to improve the transparency and consistency of risk-benefit assessments ( http://www.fda.gov/downloads/MedicalDevices/DeviceRegulationandGuidance/GuidanceDocuments/UCM296379.pdf). Recently, the FDA issued draft guidance on a structured qualitative benefit-risk framework, with several aspects of the decision making being quantitative ( http://www.fda.gov/downloads/forindustry/userfees/prescriptiondruguserfee/ucm329758).

The Institute of Medicine report on the ethics of post-marketing safety studies ( http://www.iom.edu/Reports/2012/Ethical-and-Scientific-Issues-in-Studying-the-Safety-of-Approved-Drugs.aspx) recommended that the FDA consider systematic assessment of benefit and risk during its regulatory advisory committee meetings. It recommended evaluation of the Multi-criteria decision analysis (MCDA) as one approach to group decision making during regulatory advisory committee meetings. MCDA methods are designed to help people make good decisions by helping them better understand the available information, assess their decision preferences and priorities, and enhance communication among involved stakeholders ^1– 3^. A MCDA using the Analytic Hierarchy Process (AHP; see Methods for a description) allows us to make better decisions in complex situations involving tradeoffs by explicitly considering the risks and benefits of alternatives ^4^.

The AHP is equipped to address a wide range of decisions that involve both quantitative data and additional, less-tangible input from stakeholders ^5– 8^. This is highly relevant to comparative effectiveness research as the comparison of alternative drugs or interventions is paramount. These complex situations may include tradeoffs between imperfect options, a mix of objective data and subjective options, and uncertain future outcomes ^9^.

Decisions always involve evaluative judgments; MCDA helps this process by making these judgments explicit, systematic and transparent ^1^. It also allows input from multiple stakeholders who may assign different preference weightings to the various risks and benefits. It has been used in other governmental organizational decision making ^10– 12^. Type 2 diabetes is a priority condition for comparative effectiveness research as it is a condition with multiple treatment options with multiple potential benefits and harms ^13, 14^.

As a part of the Johns Hopkins FDA Partnership in Applied Comparative Effectiveness we plan to conduct a MCDA using the AHP to determine the optimal choice of oral medications for type 2 diabetes in a simulated advisory committee setting.

## Methods

### Study population

We will invite a small group of at least eight diabetes experts from clinical (primary care, endocrinology, and pharmacy), research (epidemiology and clinical trials), operations (pharmacy and therapeutics), and public health disciplines (department of public health) related to diabetes treatment to participate. Recruitment methods will include invitations extended by email with attachment of an approved document ( Appendix A). This project received ethical approval from the Institutional Review Board of Johns Hopkins University (Approval Number: NA_00048562).

### Study intervention

The study intervention will be a four part structured interview consisting of a) overview of current medications and options for type 2 diabetes; b) a MCDA using the AHP; c) a cognitive interview; d) evaluation of the AHP-based priority assessment procedure.

### Overview of current medications and options for type 2 diabetes

We will use the most updated version of the regulatory label for the treatment options for type 2 diabetes. When data on outcomes is not available, we will use data from our Agency for Healthcare Research and Quality (AHRQ) comparative effectiveness review ^15^.

### The AHP

**Figure 1.**
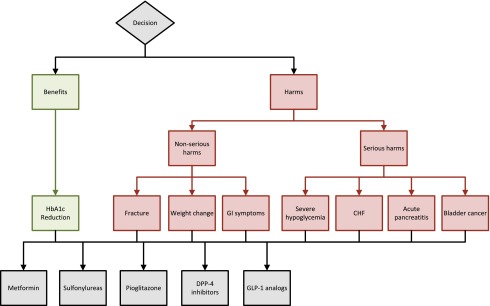
Final Analytical Hierarchy Process Model. The first step in Analytic Hierarchy Process analysis consists of defining the goal of the decision, the alternatives being considered, and the criteria to determine how well the alternatives can be expected to meet the goal. As seen above, these are organized into a hierarchical decision model with the goal at the top, alternatives at the bottom, and criteria in between.

The first step in AHP analysis involves defining the goal of the decision, the alternatives being considered, and the criteria to determine how well the alternatives can be expected to meet the goal ^5– 8^. These are organized into a hierarchical decision model with the goal at the top, the alternatives at the bottom, and the criteria in between ( Figure 1). In the second step, information about how well the alternatives can be expected to fulfill the decision criteria is collected. The third step consists of two parts: a) comparing the ability of the alternatives to fulfill the prespecified criteria, and b) judging the importance of the criteria relative to the decision goal. Pairs of alternative options are sequentially compared regarding their ability to fulfill the criteria using pairwise comparisons ^7^.

A combined normalized ratio scale summarizes the results of the direct and indirect comparisons. Priorities for alternatives are compared using ratios; relative differences of 1.1 are considered significant according to standard AHP criteria ^10^. A ratio, or relative difference, of 1.1 between two alternatives implies a 10% multiplicative difference with respect to how the alternatives meet a given objective at the next level above in the hierarchy. To measure the quality of AHP, we will measure the internal consistency of the judgments within a set of pairwise comparisons using a measure called the consistency ratio. A consistency ratio of 0 indicates perfect consistency. By convention, consistency ratios < 0.15 were considered acceptable ^7^.

Separate judgements are made for various decision perspectives. After the pairwise comparisons of alternatives, the pairwise comparison methods are used to determine the priorities of the criteria relative to the decision goal ^7^.

In the fourth step, the scales created in step three are combined to create a summary score indicating how well the alternatives can be expected to meet the decision goal ^7^.

This is similar to estimating a weighted average by multiplying the scores indicating how well the alternative options meet the decision goal by the priorities assigned to the criteria and adding the results ^7^. The priority of a given alternative with respect to meeting an objective at the next level up in the hierarchy is obtained by summing the products of the alternative weight and each objective weight at the level below in the hierarchy. The pair wise ratings will be transformed into relative weights by calculation of the right principal eigenvector of the relevant matrix (e.g., matrix of the pairwise comparisons between objectives at one level of the hierarchy). Expert Choice, uses the matrix multiplication method, considered to be accurate ^16^, for this calculation. The resulting scores are commonly expressed as percentages and indicate the alternative medications' relative abilities to fulfill the decision goal.

The fifth step consists of sensitivity analyses to explore the effects of changing the estimates or judgements used in the original analysis.

All AHP analyses will be conducted using Expert Choice 11.5 standard program. We will use the ideal synthesis mode with which rank is preserved in the case of addition or removal of an 'irrelevant' alternative ^17^. In the ideal mode, the priorities of alternatives or options at a given level of the hierarchy are divided by the priority of the highest-scoring alternative or objective (the “ideal”), and the results are weighted ^10^.

In our example, this means that the two identical highest-priority alternatives will both receive the same weight, and it will not make any difference to the weights of the other alternatives if both or only one of the irrelevant alternatives are included. In the distributive mode, priorities are divided by the sum of priorities to give a normalized weight and this allows for rank reversal ^10^. We will examine our results for robustness using the distributive mode.

We will also conduct a cognitive interview and process evaluation to evaluate the user perspectives on the process. The cognitive interview will consist of an investigator accompanying the participants as they complete the AHP task and asking a range of probing questions. Questioning will be guided by a checklist to ensure that all important aspects of the hierarchy, the choice tasks, and the instrument itself were functioning as intended. Qualitative feedback will be entered into NVivo ( http://www.qsrinternational.com/products_nvivo.aspx), coded and analyzed to identify recurring themes. Once all participants have completed the AHP, they will be invited back for a group session. At this session group results will be presented and discussed and the participants will be asked to complete an evaluation form ( Appendix B) regarding their experience with, and confidence in the AHP method. The operationalization of the AHP for our study is described below.

### Step one: Defining the decision context and creating an AHP decision model

The decision goal will be placed at the top of the hierarchy. The level of the hierarchy below the decision goal will include the general objectives. More specific objectives will be placed below the general objectives with each lower level consisting of more specific objectives. General objectives and more specific objectives at a given level of the hierarchy will be comparable. We will aim for seven or fewer objectives on any given level of the hierarchy, and objectives will be expressed positively. The pharmacologic alternatives will be placed at the lowest level of the hierarchy. The decision context, model content, and structure of the hierarchy will be validated for content and refined through in-person group sessions with experts. The panel of experts will consist of clinical experts (e.g., internists, endocrinologists, and pharmacists) and research experts (e.g., epidemiologists and clinical trialists). The decision context and hierarchical model will be presented to a panel of experts on at least two occasions. Experts will be invited to these validation sessions informally in-person or by e-mail. Sessions will be 60 to 90 minutes in duration. After each session, the expert feedback obtained will be synthesized and incorporated into a revised version of the model and decision context. During each expert session, a member of the study team will present an overview of the use of the AHP in decision-making. A list of three to five open-ended questions will be distributed in hard copy to the group for feedback on the model ( Supplementary Table 1).

The decision context will be ranking medication for type 2 diabetes. The clinical scenario presented will be an adult patient with moderately uncontrolled type 2 diabetes (glycated hemoglobin-7–9 g/dl). The stated decision goal will be to rank the options for type 2 diabetes treatment. Two criteria will be defined as determining the best treatment of type 2 diabetes: 1) to maximize benefits via glucose reduction; 2) to minimize medication adverse effects. The criteria for maximizing benefits will be focused on reducing glycated hemoglobin; the criteria for minimizing medication adverse events will be sub-divided into two sub criteria: minimizing non-serious harm and minimizing serious harm. The serious harms include severe hypoglycemia, congestive heart failure, acute pancreatitis, and bladder cancer. The non-serious harms include fractures, weight gain and gastrointestinal symptoms. The five medication options that will be considered are metformin, sulfonylureas, exenatide, sitagliptin and pioglitazone.

### Step two: assembling and organizing outcome information and presentation of the evidence matrix

We will use several sources of data for the decision-analysis. The treatment-specific probabilities or mean differences for objectives at the lowest level of the hierarchy will be identified from the FDA label or medical review documents available for each drug as available on the FDA website. In the absence of quantitative data on the specific objectives, we will substitute data from a Comparative Effectiveness Review of diabetes medications developed under contract from AHRQ ^18^.

These treatment-specific quantitative data (“evidence matrix”) will be presented relative to either the comparator identified in the decision context, or to placebo/usual care (see section on, “Presentation of objectives”).

**Supplementary Figure 1.**
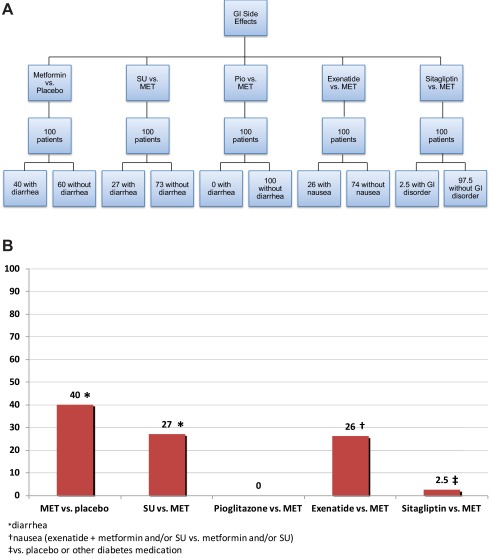
Visual representations of data. **A**) The flow chart shows the occurrence gastrointestinal (GI) side effects by diabetes medication pair. **B**) Bar chart shows excess number of patients per 100 with GI side effects. SU-Sulfonylureas; MET-Metformin; Pio-Pioglitazone.

The data from the evidence matrix will be formatted to create a visual representation which facilitates interpretation. Formats for presentation will be considered based on Dolan *et al.* ^19^ and will include display of risks on a plot with 2 axes; bar charts displaying risks; flow charts displaying risks; and pictograms that depict probabilities by displaying a box that contains 100 items, some of which are filled. These formats will be compared and discussed among the study team, and the final presentation format selected by the study team will be used to display evidence on objectives during AHP sessions. See Supplementary Figure 1 for the different visual representations to be considered.

### Step three: making comparisons among the alternatives

*Comparisons among alternative drugs relative to criteria*: We will compare the alternative drugs’ ability to achieve the decision goal (i.e. best treatment for type 2 diabetes) by making comparisons among the alternative drugs with regard to fulfilling each criterion. This will be conducted using standard AHP pairwise comparisons among the alternatives for each of the benefit and risk criteria defined in the previous step using the 9-point ratio scale. This “bottom-up approach” will be used to account for the potential consideration when one key assumption for AHP – that the higher level of elements in the hierarchy are independent of lower level elements – may not hold.

*Comparisons among the criteria:* The same pairwise comparison method will be used to determine the priorities of each of the criteria relative to the decision goal (i.e. safe and effective medication for type 2 diabetes).

### Step four: combining judgments’ to see how well alternatives can meet the goal

We will use the standard AHP weighting to combine the results of the judgments made in step three to determine the relative abilities of the medications to meet our stated goals for the decision context. Relative differences > 1.1 will be considered significant.

### Step five: sensitivity analysis

We will explore the impact of different judgments’ on the relative importance of the criteria varying their priorities from 0 (no importance) to 1 (most important) and recalculating their alternative scores. We will invite input on the relative importance of the criteria from various stakeholders and conduct additional sensitivity analysis to determine their impact on the decision goals.

***Delivery of the AHP web based instrument.*** The AHP instrument will be delivered as a series of questions in an online version of Expert Choice. The first screen, or “welcome screen”, will present a comprehensive description of the decision context, instructions as to how pair-wise comparisons should be judged using a ratio scale, and instructions for navigating through the experiment. Each subsequent screen will contain a set of pair-wise questions designed to elicit each respondent’s opinion on the relative weight of each objective or alternative in terms of meeting the overall goal or relevant objective.

Alternatives will be presented first, then specific objectives, moving from the bottom of the hierarchy up to the decision goal. A bottom-up order of presentation was considered more appropriate than a top-down order because expert respondents will have underlying knowledge about details that could influence decisions at higher levels in the hierarchy in an uncontrolled manner. For pair-wise comparisons of objectives and sub-objectives, participants will see the text: “Which of the two objectives below is more important?” For pair-wise comparisons of alternatives, participants will see the text: “Which of the two alternatives below is more preferable?” Rating of alternatives is necessary in order to transfer the evidence into subjective comparisons of importance on the ratio scale. The Expert Choice software translates all judgments to a ratio scale, regardless of whether they are entered using a verbal, graphical, or numerical scale.

The maximum number of pair-wise comparisons will be presented in order to give the best possible accuracy. Participants will be able to click on information icons to display evidence-based information (described above in “Presentation of objectives”) on how each alternative meets the relevant objective. The instructions on the welcome screen and questions will be drafted according to survey methodology best practice and will be tested and redrafted as necessary by the study team. Participants will be able to see their individual intermediate results throughout the process and their overall results when the process is completed. The option for participants to see their inconsistency ratios will be turned off because presentation of the inconsistency ratio may encourage participants to aim for consistency over accuracy. At the end of the process, participants will see a “thank you” screen which will provide a brief overview of the analysis process and group session as well as contact details for the principal investigator and ethics committee. Analysis will be conducted using the ideal mode, and various sensitivity analyses will be conducted for the group session.

### Conduct of the AHP sessions

Participants will be invited to participate in one individual session and one group session. Trained interviewers who are co-investigators on the project will schedule one-on-one appointments with respondents in which they will complete the instrument and probe questions. Interviews will take approximately 90 minutes. Once all pretests are completed, individual and group results will be analyzed and presented to respondents in a group debrief session lasting approximately 60 minutes. This will provide respondents with further opportunity to comment on the face validity of the approach and to raise any additional concerns about the instrument.

The individual interviews will involve an experienced investigator working one-on-one with each respondent to work through the AHP task and conduct the cognitive debriefing concurrently. Interviews will be scheduled at quiet locations and times convenient to the respondent and will take approximately 90 minutes to complete. Respondents will not be remunerated for their participation. Respondents will not be asked to provide any personal health information, which helps minimize the risk of breaches of confidentiality.

At the end, participants will be invited back for a group session. The objectives of the group session are: 1) to present the group results from the AHP and any significant findings from the cognitive debriefing; 2) to assess the extent to which respondents agree with the experiment’s findings; and 3) to assess whether they find the method sufficiently trustworthy to consider using it in other decision making contexts.

The group session will be held in a secure location. It will take approximately two hours, and respondents will not receive remuneration. The agenda for the group session will include presentation of overall results followed by results at each level of the hierarchy. For each trade-off in the hierarchy, presentation of results will be accompanied by discussion of any outlying results, with respondents encouraged to discuss reasons for differing views. Where there is significant heterogeneity in responses, as measured by the standard deviation of the priority weights, or where respondents do not agree with results, investigators will show the impact on results of alterations to responses and/or the evidence matrix. The session will conclude with a short poll on the extent to which respondents agree with the findings and trust the method.

### Cognitive interview and evaluation

Cognitive interviewing will be conducted alongside the individual interviews. The cognitive debriefing protocol will be developed based on standard methodology building on methods used for cognitive debriefing of conjoint analysis instruments. The protocol will involve prospective and retrospective probing, as well as assessing information volunteered by participants to assess the AHP instrument according to the following checklist shown in Table 1. Interviews will be recorded and transcribed, if agreed to by respondents, to facilitate analysis of the cognitive interviewing information. Analysis of the cognitive interview will involve assessing the extent to which the scale performed adequately on each of the checklist items. Major themes or concerns about the instrument will be elicited from the transcripts and presented through representative quotes.

**Table 1.** Checklist used to guide cognitive interviewing.

Category	Item
Validity of the objectives	Hierarchy: Do respondents understand the decision goal and objectives at each level of the hierarchy?
	Evidence: Do respondents understand the way evidence is presented?
	Information: Is there omitted information (e.g., are the questions and evidence presented sufficient)?
Choice tasks	Task understanding: Do respondents understand the task?
	Perspective: Are respondents answering from the correct perspective?
	Trade-offs: Are respondents willing to make trade-offs?
Overall survey instrument	Comprehension: Is the reading level appropriate?
	Burden: Is the respondent burden appropriate?
	Engagement: Are respondents engaged in the task?

### Study investigators

The study team will comprise of co-investigators with methodologic expertise in preference assessment methods, decision analysis, evaluation of pharmacotherapeutic published and unpublished literature, epidemiology, and clinical care of type 2 diabetes. The study team will meet for at least 45 minutes each week to discuss and refine the decision context and model development. One member of the team will take primary responsibility for development of the decision context and model based on study team discussions and feedback during validation sessions.

### Protection of human subjects

The protocol was approved by The Johns Hopkins Medicine Institutional Review Board. Risks to human subjects are minimal because the tasks involve opinion research, with no medical interventions or collection of personal health information. Participants will be provided with information about the study at the beginning of the individual sessions and asked for their consent to participate. They will be informed that they do not have to answer all questions and that they can withdraw from the study at any time without penalty.

## Discussion

We will provide the FDA with a new and innovative decision making method to ensure that potentially life-saving treatments are available to patients while protecting public health. The results from this study are likely to benefit patients and regulatory decision-makers in making judgments about the risks and benefits of drugs and devices.

Our study will provide a demonstration of the development and conduct of an AHP in the context of benefit-risk analysis that might occur in a committee setting. Using the AHP can aid, support, and enhance the understanding of decision-making processes. Cognitive interviewing will provide detailed qualitative data that could be used to improve the validity, clarity, and usability of the instrument.

Our protocol has several strengths. First, the AHP will be developed in an iterative manner that incorporates expert feedback at several steps in the process. While all participants will be experts in diabetes, each group will include some people who are new to AHP. This structure ensures that all technical considerations are addressed but also that the instrument will be as accessible as possible for people using an AHP for the first time. Second, this study will include formal process evaluations at several steps in the process, which gives structured insight into users’ perspectives on the performance of the instrument and confidence in the method. Third, we will evaluate the results of the cognitive interview to ensure confidence that the process is valid.

This will be the first study to use the AHP to rank alternatives for treatment of type 2 diabetes. The findings will be compared to other applications in the healthcare literature where the AHP has been used in a committee-style decision-making process ^20, 21^. The study will determine to what extent the optimal choice of treatment for type 2 diabetes depends on the performance of the alternatives on various benefit and harm outcomes, and the importance assigned to these outcomes.

This study will provide information on a novel comprehensive approach using the AHP to systematically address the risk-benefits in a transparent patient-centered evidence-based manner.

## Figures and Tables

**Supplementary Table 1.** Open-ended questions to obtain feedback on initial model.

**Diabetes Medication Decision Model: feedback form** Thank you for participating in the discussion about the Diabetes Medication Decision Model. In order to improve the model we would appreciate some written feedback from participants. Please take a few moments to share your thoughts about the model.
• What is your general impression of the model (e.g., structure, relevance to actual decision making)? • Was the model missing anything, and if so, what? • Was there anything in the model that should not be there and if so, what?

**Figure:**
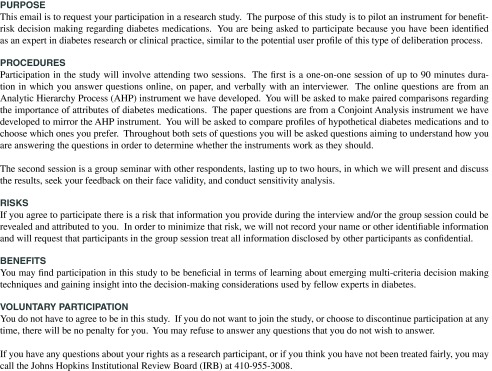


**Figure:**
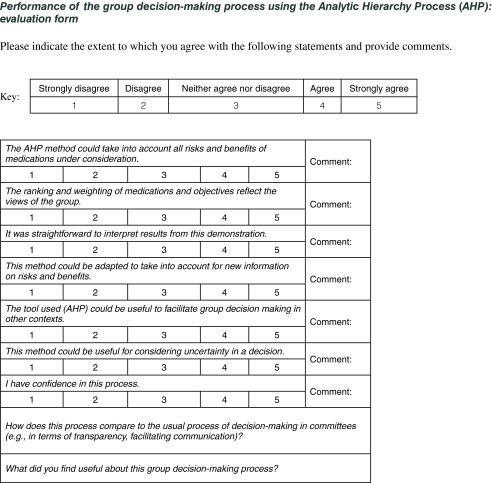


## References

[1] Belton V Stewart T: Multiple Criteria Decision Analysis: An Integrated Approach.United Kingdom: Kluwer Academic Publishers.2002 Reference Source

[2] Figueira J Greco S Ehrgott M: Multiple Criteria Decision Analysis: State of the Art Surveys.New York: Springer2005 Reference Source

[3] Dolan JG Boohaker E Allison J: Patients' preferences and priorities regarding colorectal cancer screening. *Med Decis Making.* 2013;33(1):59–70 10.1177/0272989X12453502 22895558 PMC3541437

[4] Mussen F Salek S Walker S: A quantitative approach to benefit-risk assessment of medicines - part 1: The development of a new model using multi-criteria decision analysis. *Pharmacoepidemiol Drug Saf.* 2007;16(Suppl 1):S2–S15 10.1002/pds.1435 17546573

[5] Dolan JG Isselhardt BJ Jr Cappuccio JD: The analytic hierarchy process in medical decision making: A tutorial. *Med Decis Making.* 1989;9(1):40–50 10.1177/0272989X8900900108 2643019

[6] Saaty T: How to make a decision: The analytic hierarchy process. *Interfaces.* 1994;24(6):19–43 10.1287/inte.24.6.19

[7] Singh S Dolan JG Centor RM: Optimal management of adults with pharyngitis--a multi-criteria decision analysis. *BMC Med Inform Decis Mak.* 2006;6:14 10.1186/1472-6947-6-14 16533386 PMC1431519

[8] Dolan JG: Shared decision-making--transferring research into practice: The analytic hierarchy process (AHP). *Patient Educ Couns.* 2008;73(3):418–425 10.1016/j.pec.2008.07.032 18760559 PMC2650240

[9] Dolan JG: Multi-criteria clinical decision support: A primer on the use of multiple criteria decision making methods to promote evidence-based, patient-centered healthcare. *Patient.* 2010;3(4):229–248 10.2165/11539470-000000000-00000 21394218 PMC3049911

[10] Saaty T: Fundamentals of Decision Making and Priority Theory with the Analytic Hierarchy Process.Vol VI of the AHP Series. Pittsburgh, PA: RWS Publications.2006 Reference Source

[11] Golden BL Wasil EA Harker PT: The Analytic Hierarchy Process: Applications and Studies.Berlin: Springer-Verlag.1989 Reference Source

[12] Liberatore MJ Nydick RL: The analytic hierarchy process in medical and health care decision making: A literature review. *European Journal of Operational Research.* 2008;189(1):194–207 10.1016/j.ejor.2007.05.001

[13] Singh S Loke YK Furberg CD: Thiazolidinediones and heart failure: A teleo-analysis. *Diabetes Care.* 2007;30(8):2148–2153 10.2337/dc07-0141 17536074

[14] Singh S Loke YK Furberg CD: Long-term risk of cardiovascular events with rosiglitazone: A meta-analysis. *JAMA.* 2007;298(10):1189–1195 10.1001/jama.298.10.1189 17848653

[15] Bennett WL Wilson LM Bolen S: Oral diabetes medications for adults with type 2 diabetes: An update.Agency for Healthcare Research and Quality (US); Rockville (MD).2011 21735563

[16] Saaty TL Vargas LG: Models, Methods, Concepts & Applications of the Analytic Hierarchy Process.Second ed. New York: Springer. *International Series in Operations Research & Management Science* 2012;175 10.1007/978-1-4614-3597-6

[17] Forman EH: Ideal and distributed synthesis modes for the analytical hierarchy process. The International Federation of Operations Research, Lisbon, Portugal.1993

[18] Bennett WL Maruthur NM Singh S: Comparative effectiveness and safety of medications for type 2 diabetes: An update including new drugs and 2-drug combinations. *Ann Intern Med.* 2011;154(9):602–613 10.7326/0003-4819-154-9-201105030-00336 21403054 PMC3733115

[19] Dolan JG Qian F Veazie PJ: How well do commonly used data presentation formats support comparative effectiveness evaluations? *Med Decis Making.* 2012;32(6):840–850 10.1177/0272989X12445284 22618998 PMC3515653

[20] Krishnan JA Lindenauer PK Au DH: Stakeholder priorities for comparative effectiveness research in chronic obstructive pulmonary disease: A workshop report. *Am J Respir Crit Care Med.* 2013;187(3):320–326 10.1164/rccm.201206-0994WS 23155144 PMC3603554

[21] Pecchia L Martin JL Ragozzino A: User needs elicitation via analytic hierarchy process (AHP). A case study on a computed tomography (CT) scanner. *BMC Med Inform Decis Mak.* 2013;13:2 10.1186/1472-6947-13-2 23289426 PMC3545827

