# Supplementary material for: Systematic assessment of benefits and risks: study protocol for a multi-criteria decision analysis using the Analytic Hierarchy Process for comparative effectiveness research
Source: F1000Res. 2013 Jul 24;2:160. [Version 1] doi: 10.12688/f1000research.2-160.v1 (PMC3886795; doi:10.12688/f1000research.2-160.v1)
Supplement: Peer review history

# Peer review: Referee response for version 1

**Published:** 2013-12-10

**Authors:** Saravana Kumar

**Affiliations:** 1 Division of Health Sciences, University of South Australia, Adelaide, SA, Australia

This is an interesting research study which aims to use an Analytic Hierarchy Process (AHP) in a simulated committee setting for selecting oral medications for type 2 diabetes. The study protocol is well presented and “ticks” many important methodological constructs.

However, there are areas which can be further strengthened:

1. At times, the information presented is not clear and lacks justification. For example, in page 5, the authors state that the “ *these treatment-specific quantitative data (“evidence-matrix”) will be presented relative to either the comparator...*”. What is the “evidence-matrix” and how will it be presented?
  Similarly, in step 4, the authors state that they will *“…use the standard AHP weighting to combine the results and judgements made in step three to determine relative abilities of the medications to meet our stated goals for decision contexts*”. How will the authors “combine” the “results and judgements”? What process will they use?
  Also, what does “ *meet our stated goals for decision contexts*” actually refer to? The manuscript is littered with similar statements creating ambiguity and confusion. Simple sentences with clearer explanations may be required.
   
2. Sampling – This may be buried in the manuscript, but what sampling framework will be used to identify the diabetes experts? Will it be purpose or theoretical sampling? Convenience sampling should be avoided if possible due to issues of bias.
   
3. Qualitative data analysis – How will the data collected through interviews and group sessions be analysed and reported? Will it be via content or thematic analysis? Given that this research also collects qualitative data, it is important to recognise and address rigour and trustworthiness in the research methodology.

Overall, this is an interesting and worthwhile study which could be strengthened with additional clarity and specific focus on key methodological issues. I wish the authors the best of luck with their research.

I have read this submission. I believe that I have an appropriate level of expertise to confirm that it is of an acceptable scientific standard, however I have significant reservations, as outlined above.

# Peer review: Referee response for version 1

**Published:** 2013-10-28

**Authors:** Fiath-Michael Uzoka

**Affiliations:** 1 Department of Computer Sciences and Information Systems, Mount Royal University, AB, Canada

The paper addresses a very important multi-criteria decision analysis technique (AHP), applied to an area where it is greatly needed - drug prescription. The authors present a proposal that utilizes the AHP in drug benefit-risk analysis, with the intent of submitting the research findings for consideration by the FDA.

 

The authors made efforts to elaborate on the AHP methodology. However, in the process of doing so, too many (at times unnecessary and confusing) details were presented:

    

- Figure 1: The AHP hierarchy is unclear. The decision box at the top is not indicative of the hierarchy structure associated with AHP and should be replaced by a goal box (rectangle). The diagram also presents the drugs (decision alternatives) as the lowest level of the hierarchy; that is, a sub level of all other levels. The authors need to present them as decision alternatives and not as a lower level hierarchy.
- The second to last paragraph of page 4 is unclear. What do the authors mean by “ *irrelevant alternative*”? How does the distributive mode of Expert Choice help “ *examine….results for robustness*”?
- Step 1, page 5: The authors state that “ *more specific objectives will be placed below the general objectives…*” Do they mean criteria? Why should they aim for seven or fewer objectives (criteria)?
- Step 2, page 5, final paragraph: The sentence “ *Formats for ….based on Dolan et al…box containing 100 items…*” is unclear. What is the substance of Dolan *et al*? What does the entire sentence mean? The authors need to a better explanation in this paragraph. Also Supplementary Figure A is unclear and not properly explained.
- Step 3, page 5: this is very confusing. The authors initially indicated that they would carry out a pair-wise comparison of the decision variables to determine the priority ratings of the variables yet now, in addition, the authors want to carry out a pair-wise comparison of alternatives. The alternatives are supposed to be evaluated against the global priorities.

 

In addition I have the following questions about the methodology of the study:

1. Why do the authors spend so much effort on multiple interviews and sessions with physicians, only to test the model with **one **patient (page 5, step one, second paragraph). A medical experiment of this nature (that could lead to far reaching decisions), should be tested using an adequate amount of data theerby increasing the generalisability of the model.
2. If this paper is a proposal paper (as I understand it to be), the authors do not need to discuss implementation issues such as the looks and prompts by *Expert Choice*.
3. Have the authors considered other concomitants that could affect the benefits and harms associated with a given drug? For exampple age, gender, race, and other genetic factors could contribute to the benefits and risks associated with a given drug. A fuzzy-cognitive map technique would likely produce a better result than AHP in the sense that it is a better tool for modelling cause and effect relationships.

 

In all, the idea of the study is very good. The authors need to better decide on the appropriate technique for modelling drug risk-benefit analysis, and clearly describe the technique.

I have read this submission. I believe that I have an appropriate level of expertise to confirm that it is of an acceptable scientific standard, however I have significant reservations, as outlined above.

# Peer review: Referee response for version 1

**Published:** 2013-07-30

**Authors:** M. Rashad Massoud

**Affiliations:** 1 University Research Co. LLC, Bethesda MD, USA

The article sets out to evaluate the Analytic Hierarchy Process (AHP) in a simulated committee setting for type 2 diabetes. AHP presents a new way of decision-making about drugs and devices. AHP is potentially valuable to patients and regulators as it can help weigh the risks and benefits.

I have read this submission. I believe that I have an appropriate level of expertise to confirm that it is of an acceptable scientific standard.
